# Compound Heterozygous *COX20* Variants Impair the Function of Mitochondrial Complex IV to Cause a Syndrome Involving Ophthalmoplegia and Visual Failure

**DOI:** 10.3389/fneur.2022.873943

**Published:** 2022-05-16

**Authors:** Peizheng Li, Dandan Guo, Xiufang Zhang, Kunqian Ji, Hongbo Lv, Yanli Zhang, Zhichao Chen, Jun Ma, Yaofeng Fang, Yiming Liu

**Affiliations:** ^1^Department of Neurology, Qilu Hospital, Shandong University, Jinan, China; ^2^Department of Neurology, Tianjin Medical University General Hospital, Tianjin, China; ^3^Department of Neurology, Heze Municipal Hospital, Heze, China

**Keywords:** cytochrome c oxidase 20 (COX20), mitochondrial dysfunction, whole-exome sequencings, neuropathy, ataxia

## Abstract

The cytochrome c oxidase 20 (*COX20*) gene encodes a protein with a crucial role in the assembly of mitochondrial complex IV (CIV). Mutations in this gene can result in ataxia and muscle hypotonia. However, ophthalmoplegia and visual failure associated with *COX20* mutation have not been examined previously. Moreover, the mechanism causing the phenotype of patients with *COX20* variants to differ from that of patients with mutations in other genes impairing CIV assembly is unclear. In this investigation, the aim was to assess the relation between *COX20* variants and CIV assembly. We performed detailed clinical, physical, and biochemical investigations of affected individuals. Western blotting, reverse transcription-polymerase chain reaction, and blue native-polyacrylamide gel electrophoresis were used to analyze the expression level of *COX20* and oxidative phosphorylation. A Seahorse XF Cell Mito Stress Test and enzymatic activity analysis were performed to evaluate mitochondrial function. Whole-exome sequencing revealed the same compound heterozygous mutations (c.41A > G and c.222G > T, NM_198076) in *COX20* in two siblings. This is the first description of ophthalmoplegia and visual failure associated with *COX20* variants. *In vitro* analysis confirmed that the COX20 protein level was significantly decreased, impairing the assembly and activity of CIV in patients' fibroblast. Overexpression of *COX20* using a transduced adenovirus partially restored the function of the patients' fibroblasts. Early-onset complex movement disorders may be closely related to *COX20* variants. Our results broaden the clinical phenotypes of patients with *COX20* variants showing ophthalmoplegia and visual failure. Additionally, dysfunction of *COX20* protein can impair the assembly and activity of CIV.

## Introduction

Mitochondrial complex IV (CIV) / cytochrome c oxidase (COX) as a terminal enzyme complex of the mitochondrial respiratory chain plays an important role in it, and CIV deficiency is a primary cause of oxidative phosphorylation disorders (1–3). CIV of mammals consists of 14 subunits (4–6). COX1, COX2, and COX3 subunits are encoded by the mitochondrial DNA and form the catalytic core of the enzyme. The remaining subunits are encoded by the nuclear DNA (7–10).

Cytochrome c oxidase 20 (*COX20*), also known as *FAM36A*, encodes a two-transmembrane mitochondrial inner membrane protein (11, 12) and acts as a COX2-specific assembly chaperone and stabilizes COX2 in the mitochondrial inner membrane (13). In the absence of COX20, COX2 is inefficiently integrated into CIV subassemblies, and compared with the normal situation, a decrease in enzyme activity and protein level of CIV are observed (11, 13).

Previous studies showed that *COX20* deficiency (MIM #619054) is involved in neurological disorders presenting with muscle hypotonia, ataxia, dysarthria, and axonal neuropathy (11, 14–16). However, there is a certain amount of clinical heterogeneity associated with *COX20* variants, and the mechanisms by which the genotype of our patients with *COX20* variants impairs COX assembly remain unclear. Meanwhile, like *SURF1, COA3, and COA5*, there are also several examples of mutations in structural components of CIV (1–3).

Here, we aimed to establish the relation between *COX20* variants and CIV assembly in this investigation. We also want to broaden the neurological features of *COX20* variants to reduce the misdiagnosis rate of hereditary sensory neuronopathy and expand the knowledge concerning the underlying genetic cause of these conditions.

## Materials and Methods

### Patients (II-1, Elder Sister, Age 20 Years; II-2, Young Sister, Age 6 Years)

The protocol used in this study was approved by the Human Ethics Review Board of the Qilu Hospital, Shandong University, and the families provided informed consent for their children to participate in the study.

### Exome Sequencing

We performed whole-exome sequencing of the genomic DNA isolated from the two affected siblings at Running Gene Inc. (Beijing, China). Sanger sequencing was conducted to confirm the variants and detect co-segregation with the phenotypes in the family. Furthermore, the identified genetic variants were evaluated and classified according to the American College for Medical Genetics (ACMG) standards and guidelines.

### Muscle and Nerve Biopsy

A muscle biopsy of the musculus gastrocnemius was performed on Patient II-1 at the Qilu Hospital. The normal control was considered free of neuromuscular diseases and was selected by the Neuromuscular Institute (Qilu Hospital, Shandong University) after obtaining informed consent. For examination and diagnosis, muscle biopsies (8-μm-thick) were stained with hematoxylin and eosin, modified Gömöri trichrome (MGT), nicotinamide adenine dinucleotide (NADH), succinate dehydrogenase (SDH), COX, SDH/CCO, periodic acid-Schiff, Oil Red O, and ATP.

The patient (II-1)'s nerve biopsy of the sural nerve was performed at the Qilu Hospital and the samples were stained with hematoxylin and eosin, MGT, nerve fiber, and Congo red.

### Cell Culture

The patient II-1's fibroblasts were isolated by skin biopsy at the Neuromuscular Institute of Qilu Hospital, derived from the musculus gastrocnemius under local anesthesia and cultured in Dulbecco's Modified Eagle Medium supplemented with F-12 (A4192001, Gibco) and 10% fetal bovine serum (Gibco,10099141), and 1% penicillin and streptomycin (Gibco,15140122) at 37°C in a 5% CO_2_ incubator. Human skin fibroblasts were obtained from iCell (Shanghai, China, h075).

### Adenovirus Transduction

To prove the pathogenicity of *COX20* variants, we performed functional complementation assays in patients' fibroblasts in which a WT *COX20* cDNA is re-expressed by adenovirus transduction. For adenovirus-mediated transduction, fibroblasts were seeded at 2 × 10^5^ cells per well into a six-well plate and incubated for 24 h at 37°C in a 5% CO_2_ incubator; fibroblasts at a density of 70–80% were used for further experiments. *COX20* overexpression adenovirus solution (WZ Biosciences, Shandong, China) diluted to a multiplicity of infection of 100 was added to the culture medium for 48 h, as a published protocol described (17). The overexpression efficiency was confirmed by western blotting using COX20 primary antibody (1:250, ab224570, Abcam, Cambridge, United Kingdom).

### Western Blotting

The primary antibodies were as follows: COX20 (1:250, ab224570, Abcam, Cambridge, United Kingdom), COX4 (1:1,000, 4850S, Cell Signaling Technology, Danvers, MA, United States), total OXPHOS rodent antibody cocktail (1:1,000, ab110413, Abcam), and GAPDH (1:5,000, BE3407-100, EASYBIO, Seoul, Korea) as an internal control in the muscle, along with β-actin (1:5,000, BE3212-100, EASYBIO) as an internal control in the cells. The immunoreactive signal was detected by using an ECL substrate kit (Millipore, Billerica, MA, United States). Gray values of the protein bands were analyzed with ImageJ 1.8.0r software (National Institutes of Health, Bethesda, MD, United States). Western blotting was performed independently three times to obtain comparable results.

### Blue Native-Polyacrylamide Gel Electrophoresis

Mitochondria were solubilized in a cold 1 × sample buffer (BN2003, Invitrogen) containing 2% n-dodecyl-β-D-maltoside (DDM, D4641, Sigma) and 5% G-250 Sample Additive (BN2004, Invitrogen). The lysates were centrifuged at 20,000 × *g* for 30 min at 4°C after incubation on ice for 15 min. The protein concentration was determined by the BCA protein assay (23227, Thermo Scientific), with 5 μg of protein loaded in each lane of the gel. CIV was analyzed by blue native-polyacrylamide gel electrophoresis (BN-PAGE) using a linear 4–16% gradient gel (BN1004BOX, Invitrogen) and transferred to polyvinylidene fluoride membranes by western blotting at a constant voltage of 25 V for 1 h. The membranes were then incubated in 20 ml of 8% acetic acid for 15 min to fix the proteins and blocked with 5% non-fat milk. After blocking, the membranes were incubated with primary antibodies against COX2 (1:1,000, ab15191, Abcam) and COX4 (1:1,000, ab14744, Abcam), followed by incubation with secondary antibodies from the same species. Complex II (CII) (1:1,000, ab14715, Abcam) was used as a loading control.

### Reverse Transcription-Polymerase Chain Reaction and Sanger Sequencing

Total RNA was extracted from muscles and cells using TRIzol reagent (Invitrogen,15596-026), and cDNA corresponding to the RNA was synthesized *via* reverse transcription using the cDNA Synthesis kit (GeneCopoeia, QP057). The primer sequences for reverse transcription-polymerase chain reaction (RT-PCR) were as follows: *COX20*: forward: 5′-GGTGGAGTCGCGGAGTAGTC-3′ and reverse: 5′-CACGTAAGAATGCTATCCTGG-3′. The PCR program was as follows: 94°C for 5 min; 35 cycles of 94°C for 30 s; 55°C for 30 s, 72°C for 40 s; 72°C for 5 min; hold at 10°C. The amplified products (7 μl) were separated on a 1.2% agarose gel (150 V, 30 min), using GAPDH as the internal control.

Furthermore, to detect the aberrant transcription of NM_198076.6 resulting from c.41A > G or c.222G > T mutations, the PCR products were subjected to electrophoresis in 1% agarose gels and visualized in a UV-transilluminator. The PCR product was then cloned into an empty pMD18-T vector (Takara). The generated construct was used to transform DH5α cells. The DNA isolated from individual colonies was subjected to Sanger sequencing.

### Seahorse XF Cell Mito Stress Test

The mitochondrial function of fibroblasts was measured with a Seahorse XF Cell Mito Stress Test Kit (103015-100, Agilent Technologies, Santa Clara, CA, United States) using a Seahorse XF24 Extracellular Flux Analyzer. To measure the oxygen consumption rate, mitochondrial complex inhibitors (oligomycin 1 μM, FCCP 1 μM, rotenone/antimycin A 0.5 μM) were successively added to the cell culture microplate to measure key parameters of mitochondrial function with the Seahorse XF24 Analyzer. Each sample was assayed in a minimum of three replicates and the data were normalized to the protein content in each well.

### Enzymatic Activity of Citrate Synthase and Complexes I–IV

Mitochondria were isolated from fibroblasts based on a published protocol (15) and dissolved in 250 mM mannitol and 5 mM HEPES adjusted to pH 7.4. The samples were subjected to three freeze/thaw cycles prior to enzyme assays. Mitochondrial protein at 5 μg/μl was added for the citrate synthase and complexes I, II, and IV activity reactions. Citrate synthase, a mitochondrial matrix enzyme, was used as a mitochondrial content measurement to normalize the other activities. The absorbance of the reaction solutions was measured with a Synergy H1 (Bio Tek, Winooski, VT, United States). Each sample was measured in at least triplicate.

### Statistical Analysis

All data were represented as mean ± SEM (standard error of mean) and Mann–Whitney *U* or Kruskal–Wallis tests were performed with GraphPad Prism 5.01 software (GraphPad, Inc., La Jolla, CA, United States). In all analyses, a value of *p* < 0.05 was considered to indicate significant results.

## Results

### Case Descriptions

#### Subject-1

**Figure 1:**
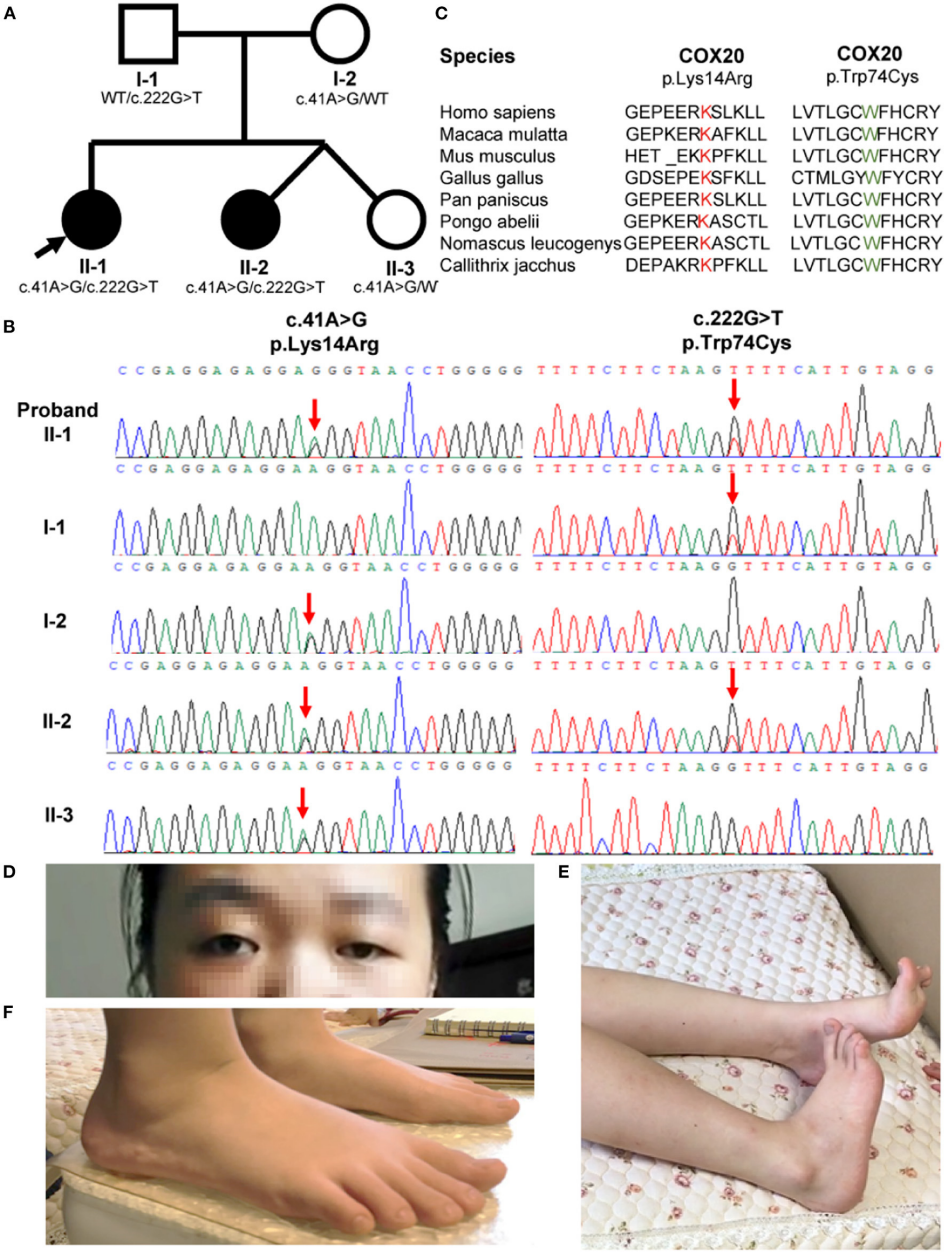
Family pedigrees and COX20 variants. **(A)** Pedigree of the COX20 family. Affected siblings are indicated in a solid circle, while asymptomatic individuals are represented in open symbols. Their genotypes are described under the symbols. Proband is indicated with an arrow. **(B)** Chromatographs of the COX20 variants of all family members. Red downward arrows point to the position of each variant. **(C)** Protein homology across divergent species. Both affected amino acid residues were highly conserved across the species. **(D)** Strabismus and left-sided ptosis (pictures from the proband II-1). **(E)** Striatal toe (pictures from the proband II-1). **(F)** Flatfoot (pictures from the proband's sister II-2).

The index patient (II-1, Figure 1A) is a 20-year-old female suffering from progressive gait unsteadiness and dysarthria. She was born by cesarean delivery to non-consanguineous parents (I-1 and I-2, Figure 1A). Developmental retardation was not suggested until the age of 4 years. She felt mild muscle soreness and weakness of the bilateral lower limbs, and strabismus was present. She began to trip and fall occasionally at 6 years, with rapid progression and confinement to a wheelchair at 10 years. At the age of 18 years, she developed progressive cerebellar dysarthria and often felt nausea in the morning, occasionally with non-ejective vomiting. Otherwise, she had no cognitive anomalies and performed well in class.

She developed lower limbs dystonia and cerebellar problems at 20 years of age. Left-sided ptosis and strabismus were observed at first sight (Figure 1D). She also had a pronounced dystonic posture of the bilateral equinovarus and striatal toe (Figure 1E). Her visual acuity in the right eye was reduced to counting fingers and to 0.15 in the left eye. Profound symmetric proximal muscle weakness of the lower limbs with 2–3/5 grades using the Medical Research Council (MRC) scale was noted. Deep tendon reflexes were absent from the lower limbers. Bilateral Babinski sign and Chaddock sign were both positive. Sensory examination showed normal results except for hyperalgesia.

Nerve conduction studies revealed severe sensory neuropathy and mild motor neuropathy. Sensory nerve action potentials of the bilateral median, sural, right radial, and ulnar could not be elicited; right motor nerve conduction velocity was decreased slightly. Somatosensory-evoked potentials could not be elicited (Supplementary Table 1). Visual-evoked potentials, magnetic resonance imaging of the brain, electrocardiography, and electroencephalography were unremarkable. Serum evaluation, including ceruloplasmin, Cu^2+^, homocysteine, lactate, creatine kinase, urine organic acid, thyroid antibodies, and cerebrospinal fluid analysis were within normal limits. Treatment with a high dose of L-DOPA was ineffective.

#### Subject-2

The proband's 6-year-old consanguineous sister (II-2, Figure 1A) developed progressive gait instability at the age of 3 years. She was a twin born by cesarean delivery. Her motor development milestone was delayed. She was unable to walk until 1.5 years and walked in a pigeon-toed manner. When running, she had to open her arms to maintain balance. Strabismus was also observed. On neurological examination at 6 years, leg dystonia was observed, and she was found to be flat-footed (Figure 1F). Her visual acuity was 0.8 in the left eye and 0.6 in the right eye. Nerve conduction studies were similar among the two patients and indicated severe sensory neuropathy. Otherwise, somatosensory-evoked potential, visual-evoked potential, fundus, serum test, and magnetic resonance imaging abnormalities were unremarkable (Supplementary Table 1). Treatment with 62.5 mg levodopa effectively improved her gait but the effect lasted for only 5 months.

Another sibling (II-3, Figure 1A) in the family and II-2 and II-3 were determined to be fraternal twins according to fluorescence short series repeat detection. This female sibling is healthy and does not show any of the symptoms described above. General physical examinations and neurological evaluations were carried out, which all showed unremarkable results.

### Nerve and Muscle Biopsy Confirm Demyelinated Neuropathy

**Figure 2:**
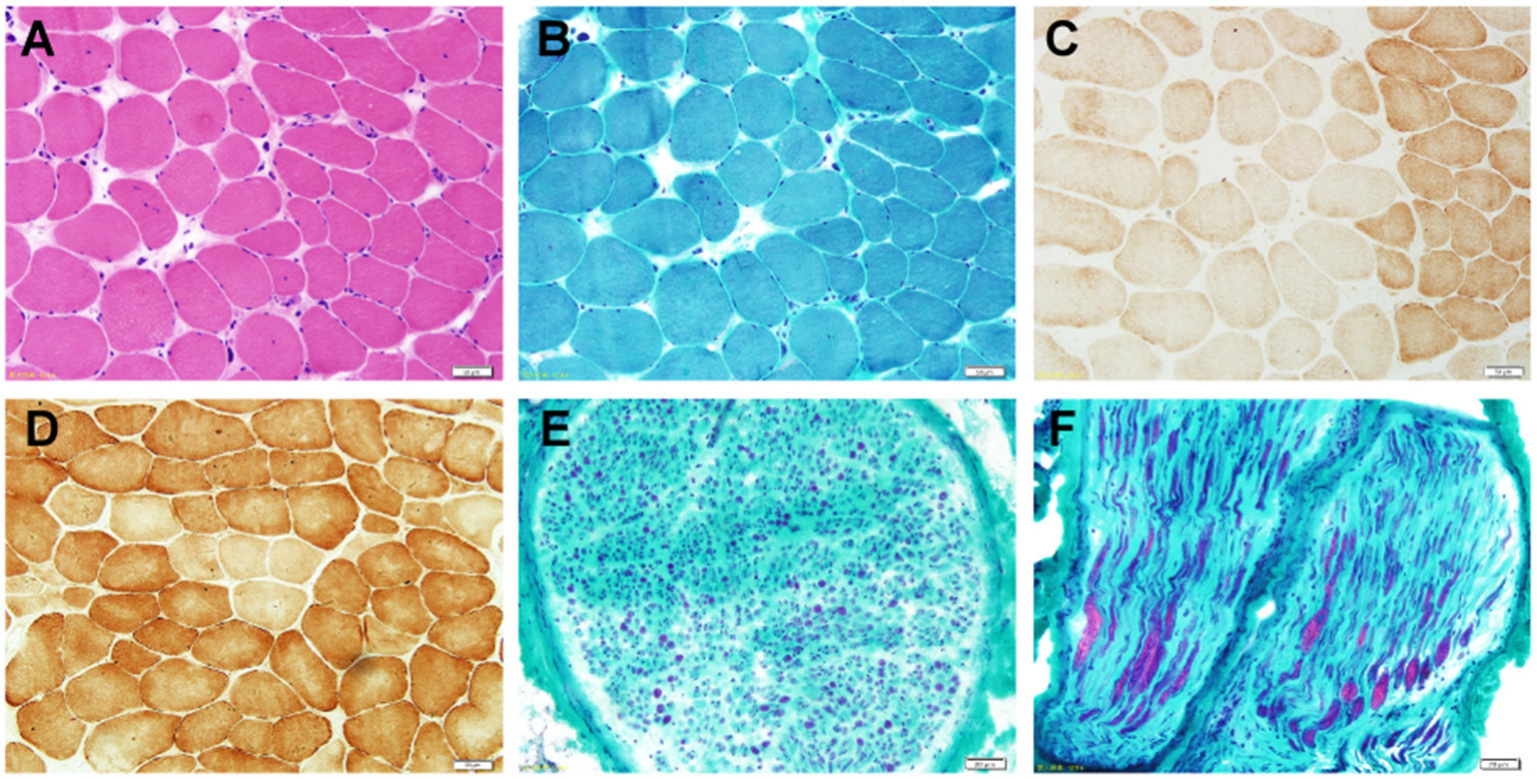
Photomicrographs of the nerve and muscle biopsy of the proband. **(A)** Hematoxylin and eosin staining (×100) shows numerous angulated atrophic fibers with an angular shape and decreased size, indicating atrophic fibers and denervation atrophy. **(B)** MGT staining (×100) reveals no ragged red fibers. **(C)** COX staining (×100) reveals extremely lower COX activity. **(D)** COX (×100) staining showed a normal muscle sample from age-matched control. **(E,F)** Pronounced depletion of large myelinated fibers by approximately 80% in the MGT-stained sural nerve (×400).

Sural nerve biopsy and muscle biopsy of the left musculus gastrocnemius were performed on the proband at the age of 20 years. Hematoxylin and eosin and MGT staining of the muscle biopsy sample revealed no ragged red fibers but showed numerous angulated atrophic fibers with an angular shape and decreased size, indicating atrophic fibers and denervation atrophy (Figures 2A,B). COX staining revealed extremely lower COX activity of the muscle fibers from the proband, compared to a normal muscle sample from age-matched control (Figures 2C,D). Large myelinated fibers were prominently decreased by approximately 80% in MGT staining, reflecting severe demyelinating neuropathy. We observed no myelin digestion chamber, onion-bulb formation, significant inflammatory cell infiltration, or edema (Figures 2E,F). Staining with SDH, SDH/CCO, NADH, CCO, periodic acid-Schiff, Oil Red O, and Congo red in the muscle specimens from the proband revealed normal or nonspecific findings.

### Exome Sequencing Analysis

Whole-exome sequencing analysis was performed on the genomic DNA from the two affected siblings. Exome sequencing revealed two compound heterozygous variants of the *COX20* gene (c.222G > T and c.41A > G) that co-segregated with the disorder in both patients. The same variants were recently reported in a patient with ataxia and muscle hypotonia (15). The asymptomatic parents and sister of the patients were heterozygous carriers, following the characteristics of family segregation (Figure 1B). The unaffected father and mother carried c.222G > T and c.41A > G (NM_198076), respectively. The affected amino acid residues were highly conserved across the species (Figure 1C). One of the alleles contained the c.222G > T mutations, which led to the missense mutation p.Trp74Cys. in the protein. The p.Trp74Cys. was predicted to be pathogenic by SIFT (predicted score: 0, damaging), Polyphen-2 (HDIV: 1; HVAR: 0.998, both probably damaging) and MutationTaster2 (predicted score: 1, disease-causing). Additionally, the mutation occurred in early exonic positions and is predicted to affect protein conformation by forming a new β-sheet according to MutPred (http://mutpred.mutdb.org/). Briefly, c.222G > T (14) was considered to be potentially pathogenic according to the ACMG standards and guidelines (likely pathogenic = PS1 + PS3 + PM2 + PM3) (18, 19). The other allele contained the single nucleotide change c.41A > G and led to the missense mutation, p.Lys14Arg. This variant was predicted to be benign by SIFT (predicted score: 0.135, tolerant), Polyphen-2 (HDIV: 0.005, HVAR: 0.018, both benign), MutationTaster2 (predicted score: 0.831285, disease-causing), and MutPred (predicted score: 0.149). The mutation c.41A > G (14, 15, 20) was classified as likely pathogenic according to the ACMG standards and guidelines (likely pathogenic = PS1 + PS3 + PM2 + PM3). In addition, genetic tests for SCA1, 2, 3, 6, 7, 12, and 17, and dentatorubral-pallidoluysian atrophy (DRPLA) of CAG repeat expansion were negative.

### *COX20* Expression in Muscle Tissue and Fibroblasts Was Lower in the Proband Than in the Control

**Figure 3:**
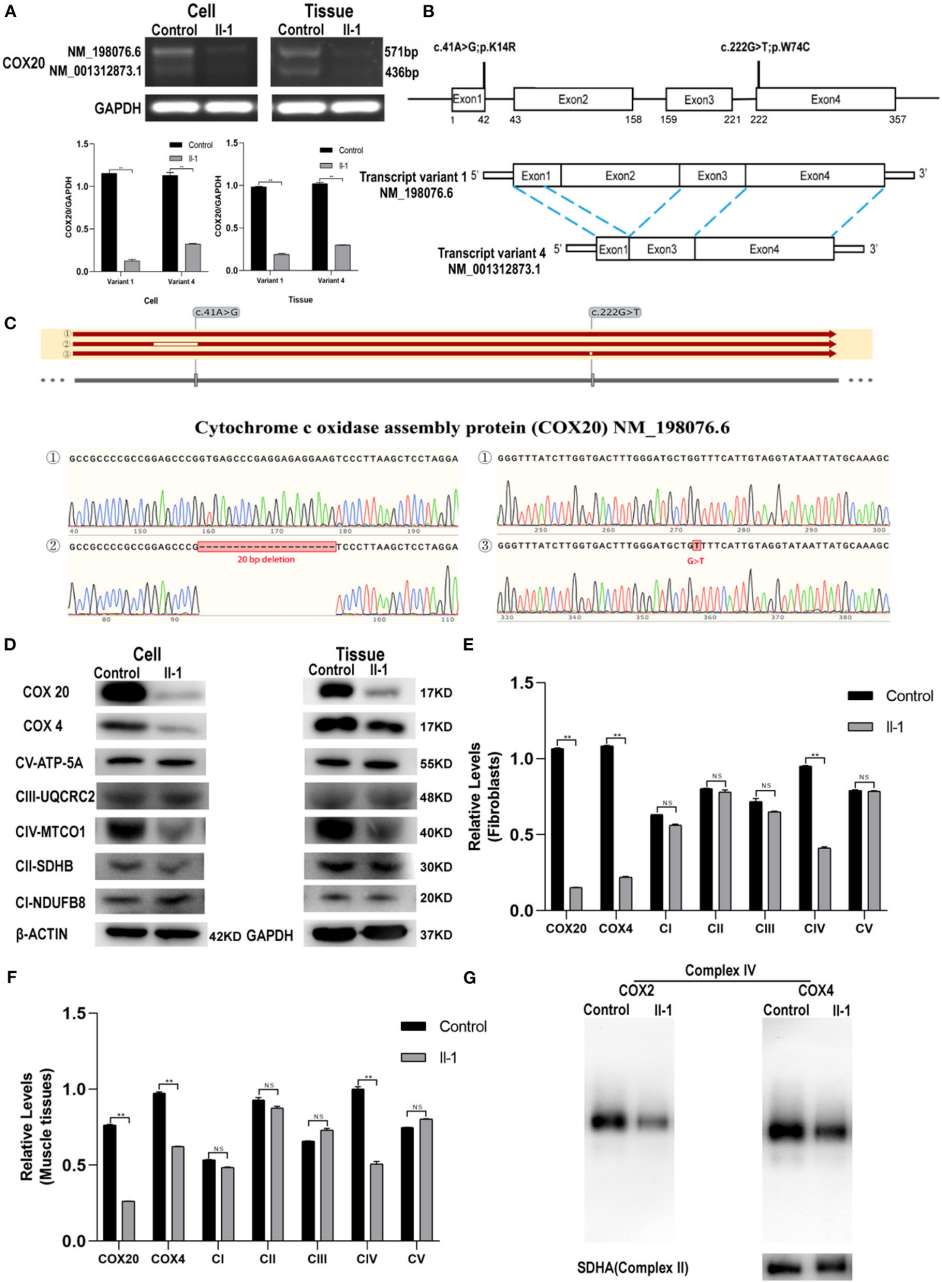
COX20 mRNA and protein expression. **(A)** mRNA expression of *COX20* in the muscle tissues and fibroblasts of the proband and control. *GAPDH* was used as the loading control. Significant differences in the mRNA levels exist between the groups. **(B)** Gene and cDNA schematic. c.41A > G and c.222G > T mutations were identified in the affected siblings. According to an NCBI genome analysis, the full length cDNA corresponds to transcript variant 1 (NM_198076.6), and the coding sequence (CDS) is 357 bp in length. Transcript variant 4 (NM_001312873.1) lacks exon 2 and a part (20 bp) of exon 1 of NM_198076.6. The coding region is 222-bp long and shorter than the transcript variant 1 by 135 bp. **(C)** Sanger sequencing reveals errors in mRNA splicing in fibroblasts of the patient. The results of sequences ①/②/③ were aligned with the reference sequence NM_198076.6. Blanks in the sequence alignment depict deletion or mismatch of nucleotides. Sequences ① were obtained from fibroblasts of control. Alignment of sequence ② shows that the variant c.41A > G led to a 20-bp deletion in exon 1. Alignment of sequence ③ shows that the variant c.222G > T did not affect mRNA splicing. **(D–F)** Western blot analysis of COX4, COX20, and OXPHOS in muscle tissues and fibroblasts. GAPDH was used as the internal control for muscles, and β-actin for cells. Significant differences are observed in the protein expression of COX20 and CIV but not in the protein expression of CI, CII, CIII, and CV. **(G)** Blue native-PAGE analysis of mitochondria isolated from fibroblasts reveals lower COX2, COX4, and CIV levels; **p < 0.01. All data are presented as mean ± SEM and *p*-values were calculated by the Mann–Whitney *U*-test.

To explore the effect of *COX20* deficiency, we tested the mRNA and protein expression of *COX20* in both the muscle tissue and fibroblasts of the proband. RT-PCR analysis showed two distinct transcript variants: transcript variant 1(NM_198076.6), which was the longest transcript, and transcript variant 4 (NM_001312873.1) (Figure 3B), lacking exon 2 and a part (20 base pairs) of exon 1 of NM_198076.6 (Figure 3B). Compared with those in the control, the mRNA levels of *COX20* in the fibroblast and muscle tissue of the patient were significantly diminished (Figure 3A). TA clone sequencing demonstrated that aligned with the reference sequence (NM_198076.6), the c.41A > G mutation led to a 20 bp deletion in exon 1. Interestingly, the c.222G > T mutation did not affect the mRNA splicing (Figure 3C). This indicated that the variant c.41A > G caused aberrant splicing and resulted in a premature stop codon (TGA) Gly8ValfsTer2, which created a shorter, unstable transcript, leading to nonsense-mediated mRNA decay (20).

COX20 protein expression in the patient's fibroblast and tissue was notably decreased compared with that in the control (Figures 3D–F). Then, the variants might make COX20 protein unstable, causing it to become easily degraded. To evaluate other adverse effects due to the *COX20* variants, we investigated COX4 and the oxidative phosphorylation electron transport chain complex subunit levels by western blotting. Compared to the control, we observed significant differences in the levels of CIV, but not in CI, CII, CIII, and CV (Figures 3D–F). It also demonstrated the variants could affect the steady-state levels of assembled CIV.

### *COX20* Variants Affect the Complex IV Assembly Process

BN-PAGE was performed to further confirm whether the *COX20* variants affect the complex IV assembly process. The BN-PAGE analysis showed lower levels of COX2 and COX4, which are individual complex IV subunits, compared to CII as a loading control. These findings demonstrate that *COX20* variants led to a specific reduction of CIV levels and very low residual levels of fully assembled CIV (Figure 3G).

### *COX20* Variants Impair Mitochondrial Function

**Figure 4:**
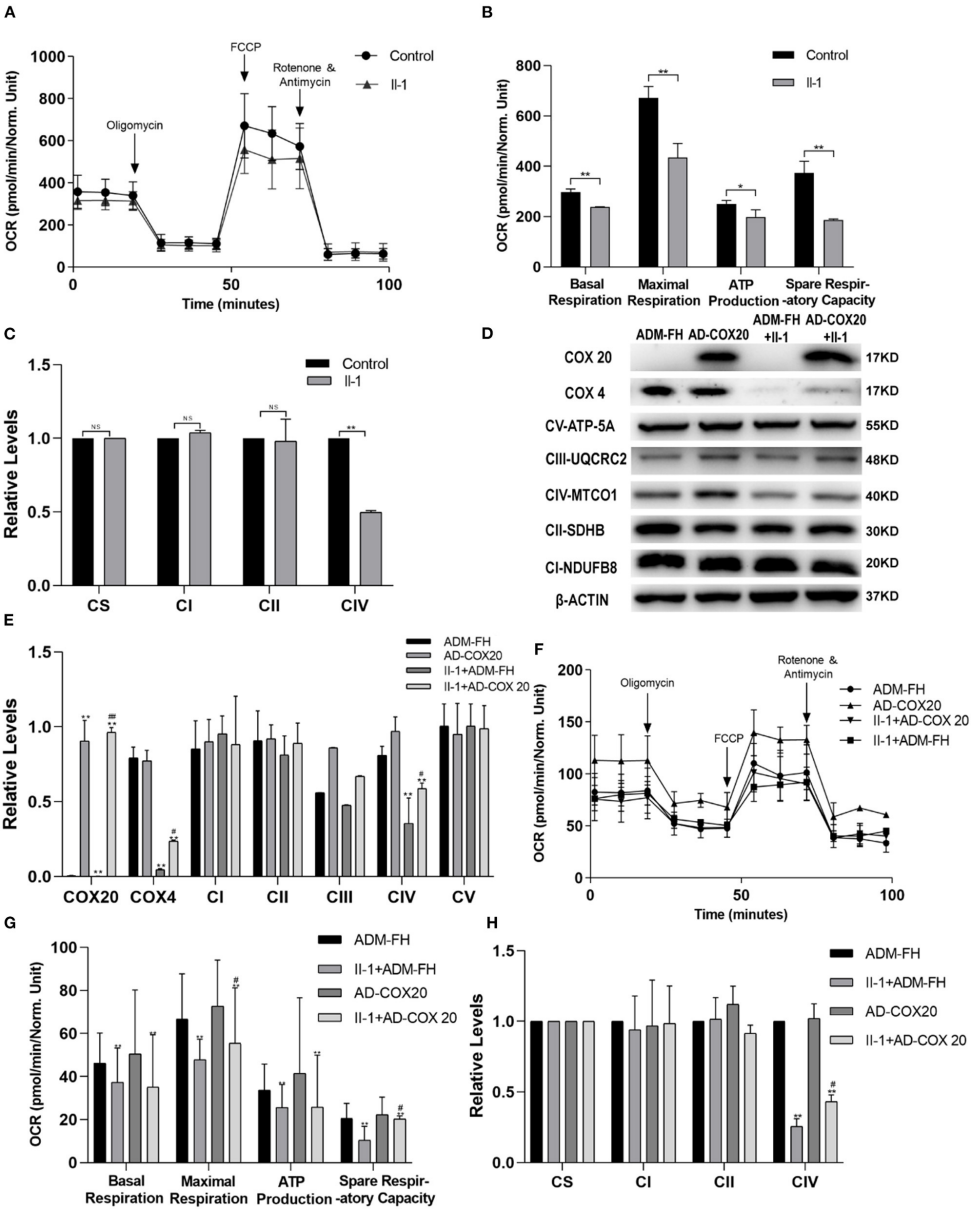
**(A,B)** OCR of patient (II-1) fibroblasts was measured using a Seahorse XF24 Extracellular Flux Analyzer. Basal respiration, maximal respiration, ATP production, and spare respiratory capacity of fibroblasts from the patient are significantly lower than those of control. **(C)** To detect the enzymatic activities of complexes, mitochondria were isolated from fibroblasts, and citrate synthase was used as the internal control. Complex IV enzyme activities of the patient fibroblasts were lower than control. **(D,E)** Functional complementation assays demonstrate that COX20 variants are responsible for the deficiency of complex IV and mitochondria in patient fibroblasts. Fibroblasts transduced with adenovirus vector (ADM-FH) were used as a control group. Western blot analysis shows that compared with fibroblasts transduced with ADM-FH, the expression level of COX20 and CIV in fibroblasts from patients transduced with COX20 increased significantly. **(F,G)** Seahorse analysis shows that COX20 overexpression promoted maximal respiration and increased mitochondrial spare respiratory capacity. **(H)** Enzyme activity measurements show that COX20 overexpression can help mitochondria restore the enzymatic activity of complex IV; *p < 0.05, **p < 0.01 vs. ADM-FH, ^#^*p* < 0.05 vs. II-1 + ADM-FH and II-1 + AD-COX20. All data are presented as mean ± SEM and *p*-values were calculated by Kruskal–Wallis tests. ^##^*p* < 0.01 versus II-1+ADM-FH and II-1+AD-COX20.

The mitochondrial function of the fibroblasts was evaluated by measuring the oxygen consumption rate using a Seahorse XF24 Extracellular Flux Analyzer. The oxygen consumption rate of fibroblasts derived from the patient (II-1) was significantly lower than that of control skin fibroblasts, as evidenced by the basal respiration, maximal respiration, ATP production, and spare respiratory capacity levels. These data suggest that *COX20* variants impair oxidative phosphorylation and mitochondrial function (Figures 4A,B).

### *COX20* Variants Impact Enzyme Activity of Mitochondrial Respiratory Chain Complex IV

The activities of complexes I–IV were expressed as a ratio of the rate of citrate synthase.

The results indicated that the enzyme activities of the mitochondrial respiratory chain complexes significantly differed between the control and the patient. The enzyme activities of complex IV were lower in the patient's cells than in control cells (Figure 4C).

### Adenovirus Transduction of Patient Cells With *COX20* Restores Cell's Partial Function

To further demonstrate that *COX20* variants are responsible for the defective assembly of CIV and mitochondrial function impairment in the patient's fibroblasts, we performed functional complementation assays by transducing *COX20* overexpression adenovirus into the patient's fibroblasts. The fibroblasts transduced with the adenovirus vector (ADM-FH) were used as a control group. Western blot analysis showed that compared to fibroblasts transduced with ADM-FH, the expression of COX20 and CIV subunits protein in fibroblasts from patients transduced with *COX20* increased significantly. However, *COX20* overexpression adenovirus does not affect the protein levels of other respiratory complexes (Figures 4D,E). Assessed by Seahorse XF24 Extracellular Flux Analyzers, *COX20* overexpression promoted maximal respiration and increased spare respiratory capacity of the mitochondria (Figures 4F,G). Consistent with these observations, the OXPHOS enzyme activity measurements showed that *COX20* overexpression can help the mitochondria restore the enzyme activity of complex IV (Figure 4H). These data supported the fact that dysfunctional COX20 protein can impair the assembly and activity of complex IV.

## Discussion

In this study, we provided genetic, biopsy, functional evidence, and functional complementation assays to support that the *COX20* gene is a novel pathogenic gene. Compound heterozygous mutations (c.41A > G and c.222G > T, NM_198076) in the *COX20* gene were identified in two siblings from a non-consanguineous family presenting with ataxia, dystonia, ophthalmoplegia, dysarthria, and sensory-dominant neuropathy. Ophthalmoplegia and visual failure associated with *COX20* variants have not been described previously in patients with sensory-dominant neuropathy. The variants decreased the level of *COX20* and CIV subunits affected the assembly of CIV and impaired the function of the mitochondria.

The function of *COX20* has been widely studied. Szklarczyk et al. found that *COX20* can promote and stabilize mature unassembled COX2 to enable it to function in the early steps of complex IV assembly (11, 15). Bourens et al. confirmed that *COX20* cooperates with SCO1 and SCO2 to complete the formation of the copper-containing redox center present in COX2 by protein pull-down and immunoprecipitation analyses (13). *COX20* variants were predicted to be pathogenic and extremely rare variants in public databases. Four potential pathogenic variants related to *COX20* genes have been reported, including a homozygous mutation (c.154A > C) (11) and two heterozygous mutations (c.41A > G and c.157 + 3G > C; c.41A > G and c.222G > T) (9, 10) (Figure 3B). Patients with *COX20* variants showed clinical symptoms during childhood and gradually developed additional signs such as dystonia, dysarthria, and sensory-dominant neuropathy. Cognitive impairment, psychiatric disorder, attention-deficit hyperactivity syndrome, and static encephalopathy have also been reported in a few cases (Supplementary Table 2) (11, 14–16, 20).

However, visual failure, strabismus, and drooping eyelid, which were observed in our patients, have not been described previously. Ophthalmoplegia and visual failure are hallmarks of these sibling patients and were not previously reported as associated with *COX20* deficiency. Numerous variants have been reported to cause ophthalmoplegia and visual failure in COX-related disorders. Early-onset COX deficiency can be traced to mutations in assembly factors, which are encoded by nuclear genes (21). These nuclear mutations lead to a diverse spectrum of overlapping phenotypes. For example, frequent symptoms of the Leigh syndrome associated with COX deficiency include hypotonia, poor feeding/vomiting, developmental delay, oculomotor involvement, central respiratory failure, and optic atrophy (22). Optic atrophy and external ophthalmoplegia may be the major phenotypes of patients with COX deficiency (23). In the optic nerve, the unmyelinated prelaminar and laminar regions are rich in mitochondria because of their high-energy requirements for electrical conduction (24, 25). Thus, the optic nerve is vulnerable to subtle changes in mitochondrial function. This explains why optic atrophy is one of the major phenotypes of primary inherited mitochondrial diseases, such as Leigh syndrome (25).

Sensory-dominant axonal neuropathy was previously reported as a prominent feature of *COX20* deficiency (20). In addition, Friedreich ataxia, SCA4, SCA25, and some mitochondrial diseases such as myoclonic epilepsy with ragged red fibers exhibit symptoms of ataxia and sensory neuropathy. Nerve conduction analysis revealed sensory neuropathy and preserved motor nerve conduction in our cases as per demyelinated pathology. However, we did not detect symptoms of dysesthesia except for hyperalgesia. The possibility was sensory nerve conduction limited to the evaluation of myelinated peripheral nerve fibers with large axon diameters. Pain and temperature measurements are conducted for myelinated small fibers and unmyelinated fibers. As a result, when the pathology is confined to large myelinated nerve fibers, pain and temperature remain normal in patients. Another possibility was that sensation analysis may be unreliable in patients with poor coordination.

However, patients with *COX20* variants also showed numerous differences in their phenotypes. This may be explained by the following: (1) Patient phenotypes are affected not only by genotypes but also by families and the environment (26). (2) There may be other modifying genes that have not been identified (27). (3) *COX20* variants, which cause mitochondrial disease, may exhibit a threshold effect and individual differences (28).

We further proved the pathogenicity of *COX20* variants by functional experiments. We found that the expression of COX20, COX4, and OXPHOS CIV subunit, and COX1 were decreased significantly because of the heterozygous mutations in the *COX20* gene. OCR measured by Seahorse XF and enzymatic activity showed mitochondrial damage. Consistent with these findings, overexpression of *COX20* in the patient's fibroblasts could partially restore this function. These results meant that *COX20* variants affected the assembly process of CIV, as demonstrated by Bourens et al. (13).

In summary, we identified pathogenic compound heterozygous *COX20* variants leading to decreased *COX20* expression and impaired assembly and activity of complex IV. Our study broadens the clinical phenotypes of ophthalmoplegia and visual failure associated with *COX20* variants. However, as this study was limited to a single family, additional cases should be evaluated to confirm our results.

## Data Availability Statement

The datasets presented in this study can be found in online repositories. The names of the repository and accession number(s) can be found below: NCBI SRA, accession numbers: SRR18749879 and SRR18749880. The raw data supporting the conclusions of this article will be made available by the authors, without undue reservation.

## Ethics Statement

The studies involving human participants were reviewed and approved by the Ethics Committee of Qilu Hospital of Shandong University. The patients/participants provided their written informed consent to participate in this study. Written informed consent was obtained from the individual(s) for the publication of any potentially identifiable images or data included in this article.

## Author Contributions

PL and YL conceived and designed the research and wrote the manuscript. PL and DG conducted the experiments with guidance of KJ. YZ, YF, and XZ performed the data collection and helped complete the statistical analysis. PL, DG, and HL performed data interpretation and the literature search. HL, ZC, JM, and KJ offered helpful guidance for this study. All authors have read and approved the manuscript.

## Funding

This work was supported by grants from the National Natural Science Foundation of China (grant no. 81771373).

## Conflict of Interest

The authors declare that the research was conducted in the absence of any commercial or financial relationships that could be construed as a potential conflict of interest.

## Publisher's Note

All claims expressed in this article are solely those of the authors and do not necessarily represent those of their affiliated organizations, or those of the publisher, the editors and the reviewers. Any product that may be evaluated in this article, or claim that may be made by its manufacturer, is not guaranteed or endorsed by the publisher.
