# Supplementary material for: Compound Heterozygous COX20 Variants Impair the Function of Mitochondrial Complex IV to Cause a Syndrome Involving Ophthalmoplegia and Visual Failure
Source: Front Neurol. 2022 May 16;13:873943. doi: 10.3389/fneur.2022.873943 (PMC9149563; doi:10.3389/fneur.2022.873943)
Supplement: Supplementary file 1 [file Table_1.docx]

Supplementary table 1. Electrophysiological studies of two patients (II-1 and II-2)

| Sensory nerves | Proband (II-1) | |  | Younger sister (II-2) | |
| --- | --- | --- | --- | --- | --- |
|  | SNCV (m/s) | SNAP (μV) |  | SNCV (m/s) | SNAP (μV) |
| L median | NE | 1.15🡫 71.2% |  | 36.1🡫 27.8% | 4.36 |
| R median | NE | 23.6 |  | 36.1🡫 27.8% | 4.81 |
| L ulnar | 34.6🡫 30.8% | 1.87🡫 53.4% |  | 42.1🡫 15.8% | 8.30 |
| R ulnar | NE | 143.2 |  | 50.9 | 6.85 |
| L superficial peroneal | NE | 102.0 |  | 56.0 | 2.90 |
| R superficial peroneal | NE | 260.9 |  | 63.7 | 1.93🡫 3.3% |
| L sural | NE | 537.2 |  | 37.5🡫 6.3% | 6.80 |
| R sural | NE | 472.1 |  | 43.2 | 7.89 |
| Motor nerves | MNCV (m/s) | CMAP (mV) |  | MNCV (m/s) | CMAP (mV) |
| L median | 53.6 | 6.83 |  | 46.3🡫 7.4% | 3.54🡫 11.6% |
| R median | 58.3 | 8.30 |  | 49.5🡫 1.0% | 4.12 |
| L ulnar | 55.2 | 7.09 |  | 55.3 | 6.89 |
| R ulnar | 57.0 | 8.85 |  | 55.0 | 7.20 |
| L deep peroneal | 47.6 | 1.06🡫 47.0% |  | 46.0 | 1.20🡫 48.2% |
| R deep peroneal | 48.8 | 4.10 |  | 47.5 | 4.32 |

Amp. amplitude, CMAP compound muscle action potential, Dur. duration, MNCV motor nerve conduction velocity, ND no data, NE not evoked, SNCV sensory nerve conduction velocity, SNAP sensory nerve action potential
