# Supplementary material for: Compound Heterozygous COX20 Variants Impair the Function of Mitochondrial Complex IV to Cause a Syndrome Involving Ophthalmoplegia and Visual Failure
Source: Front Neurol. 2022 May 16;13:873943. doi: 10.3389/fneur.2022.873943 (PMC9149563; doi:10.3389/fneur.2022.873943)
Supplement: Supplementary file 2 [file Table_2.docx]

Supplementary table 2. Summarized and analyzed COX20 variants

| Studies | Country of origin |  | Mutations | | |  | Patients and age of onset (Y, years) | Phenotype | Biochemical experiments |
| --- | --- | --- | --- | --- | --- | --- | --- | --- | --- |
|  |  |  | DNA | Protein | State |  |  |  |  |
| Present study | China |  | c.41A>G  c.222G>T | p.Lys14Arg  p.Trp74Cys | Heterozygous |  | Elder sister(4Y)  Little sister(4Y) | ataxia, dystonia, sensory-dominant neuropathy and ophthalmoplegia | 1.RT-PCR: the expression of COX20 mRNA decreased in fibroblasts;  2. TA clone sequencing：the variant c.41A>G caused aberrant splicing and resulted in a premature stop codon (TGA) Gly8ValfsTer2.  3.Western Blot: the expression of COX20, COX4, CIII and CIV proteins decreased in muscle and fibroblasts;  4.OXPHOS enzyme activity: enzyme activities of CIII and CIV decreased;  5.Seahorse XF cell Mito Stress test: mitochondrial function impaired. |
| Szklarczyk et al. (2013) | German |  | c.154A＞C | p.Thr52Pro | Homozygous |  | / | cerebellar ataxia, hypotonia, and delayed speech development | 1.BN-PAGE: the function of complex IV decreased in fibroblasts;  2. Western blot: the expression of COX20, COX1, COX2, COX4 and COX5A protein decreased;  3.QPCR: no difference;  4.OXPHOS enzyme activity: severe complex IV deficiency in muscle and fibroblasts. |
| Doss et al. (2014) | Turkey |  | c.154A＞C | p.Thr52Pro | Homozygous |  | Proband(12Y)  Elder sister (5Y) | cerebellar ataxia, dystonia, torticollis and sensory axonal neuropathy | 1.RT-PCR: the expression of COX20 mRNA decreased in fibroblasts;  2. Morphology of mitochondrial network: normal, increased fusion in mutant cells;  3. HPLC and enzyme activity: CIV and a coenzyme Q10 deficiency in muscle biopsy. |
| Otero et al. (2019) | America |  | c.41A>G  c.340G>A  c.157+3G>C | p.Lys14Arg  p.Gly114Ser | Heterozygous |  | From 17 months to 2 years | hypotonia, ataxia, dystonia, dysarthria, attention-deficit hyperactivity syndrome psychiatric disorder and sensory neuropathy | 1.RT-PCR: the expression of COX20 mRNA decreased in fibroblasts;  2.Western blot: no COX20 expression in fibroblasts. |
| Hongliang et al. (2019) | China |  | c.41A>G  c.222G>T | p.Lys14Arg  p.Trp74Cys | Heterozygous |  | Proband (1Y)  Little brother (3Y) | sensory-dominant neuropathy, dysarthria, intellectual disability and static encephalopathy | 1.Sural nerve biopsy: morphological alternations of mitochondria and axonal neuropathy;  2.Western blot: the expression of COX20 protein decreased in leucocytes. |
|  |  |  |  |  |  |  |  |  |  |
